# Deleted in malignant brain tumor *1* genetic variation confers urinary tract infection risk in children and mice

**DOI:** 10.1002/ctm2.477

**Published:** 2021-07-04

**Authors:** David S. Hains, Shamik Polley, Dong Liang, Vijay Saxena, Samuel Arregui, John Ketz, Evan Barr‐Beare, Ashley Rawson, John D. Spencer, Ariel Cohen, Pernille L. Hansen, Martina Tuttolomondo, Cinzia Casella, Henrik J. Ditzel, Daniel Cohen, Edward J. Hollox, Andrew L. Schwaderer

**Affiliations:** ^1^ Department of Pediatrics Indiana University Indianapolis Indiana USA; ^2^ Department of Genetics and Genome Biology University of Leicester Leicester UK; ^3^ The Center for Clinical and Translational Research The Research Institute at Nationwide Children's Hospital Columbus Ohio USA; ^4^ Brown University Providence Rhode Island USA; ^5^ Lundbeckfonden Center of Excellence NanoCAN University of Southern Denmark Odense Denmark; ^6^ Department of Cancer and Inflammation Research, Institute of Molecular Medicine University of Southern Denmark Odense Denmark; ^7^ Department of Oncology Odense University Hospital Odense Denmark; ^8^ Emergency Department Nationwide Children's Hospital Columbus Ohio USA

Dear Editor,

We identify that children with a history of urinary tract infections (UTIs) and vesicoureteral reflux (VUR) with low deleted in Malignant Brain Tumors 1 (*DMBT1*) DNA copy number have ∼4‐fold higher odds of recurrent UTIs compared to those with high *DMBT1* DNA copy number. With bacterial resistance to antibiotics increasing, we need to develop new strategies to guide judicious use of antibiotics such as using the patient's genetic profile.^1^

Some groups, such as children with VUR, are at risk for UTIs and associated adverse outcomes. The Randomized Intervention for Children with VUR (RIVUR) study determined that antibiotic prophylaxis reduced recurrent UTIs in children with VUR but at the expense of increased resistance.^2^ In a genome‐wide array, we identified that *DMBT1* locus was a candidate gene for genetic variation in the UTI/VUR patient population.^3^ We have also shown that *DMBT1* has two copy number variations which occur in scavenger receptor cysteine‐rich domains (SRCR) which have bacterial agglutination capabilities.^4^, ^5^ The first CNV involves SRCR3–SRCR6 and is called *DMBT1* CNV_SRCR3‐6_; the other involves SRCR9–SRCR11 and is called *DMBT1* CNV_SRCR9‐11_ (Supplemental Material S1).^4^

We used paralogue ratio testing to determine the absolute *DMBT1* copy number in RIVUR patients. We also evaluated the biological relevance of *DMBT1* in experimental UTI models of a knockout mouse (*Dmbt1* ^−/−^).^6^ Agglutination studies of uropathogenic *Escherichia coli* (UPEC) with recombinant DMBT1 6 kb (DMBT1^gp340^‐short) or recombinant DMBT1 8 kb (DMBT1^gp340^‐long) as surrogate model for the protein produced by low and high *DMBT1* copy number respectively were also performed.^7^ Full details about the methods may be found in Supplemental Material S2.

We analyzed DNA on 301 European‐American females from the RIVUR study compared to 266 race, ethnicity, and gender matched controls with no known UTI or VUR history. The RIVUR subjects were younger than the controls with median interquartile range ages of 1.3 (0.7–2.9) versus 11.7 (8.0–14.7) years, respectively, *p* < 0.001. Because our RIVUR cohort was randomized to placebo (*n* = 148) or antibiotic prophylaxis (*n* = 153) and followed for 2 years for UTI recurrence, we were able to analyze the two treatment groups separately. The age in years was not statistically different between treatment groups at 1.3 (0.7–3.0) and 1.4 (0.7–2.9), respectively.

*DMBT1* copy number was determined in 289 of 301 RIVUR patients along with 266 and 247 control patients for CNV_SRCR3‐6_ and CNV_SRCR9‐11_, respectively. We did not find a difference in *DMBT1* DNA copy number distribution between the whole RIVUR cohort and controls (Supplemental Material S3). In patients treated with placebo, we found that patients with low *DMBT1* CNV_SRCR9‐11_ copies, based on the median copy, had ∼4 times the odds of experiencing recurrent UTIs during the study period compared to individuals with a higher copy number (Figure 1). In the antibiotic prophylaxis randomized group, *DMBT1* CNV_SRCR9‐11_ copy number in this region was not associated with differences in recurrent UTI risk suggesting that the genetic predisposition can be overcome with antibiotic prophylaxis.

**FIGURE 1:**
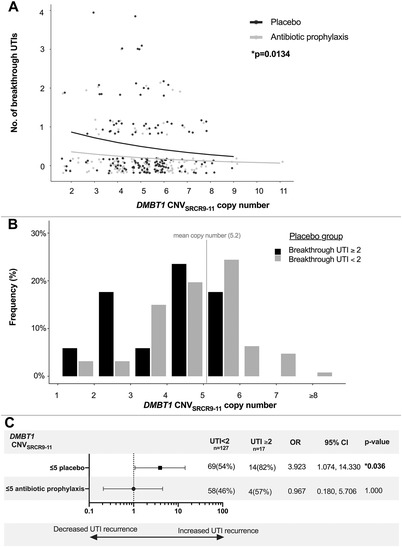
Low copy number of *DMBT1* CNV_SRCR9‐11_ is associated with recurrent UTIs in children with VUR. (A) In children with VUR and high *DMBT1* CNV_SRC9‐11_ copy number, the number of recurrent UTIs is similar whether the patients are treated with placebo (black line) or antibiotic prophylaxis (gray line). In patients on placebo (black line), the number of breakthroughs UTIs increases as DMBT CNV SRC‐9‐11 decrease the number of recurrent UTIs increases. In comparison, the recurrent UTI rate remains relatively constant in patients treated with antibiotic prophylaxis (gray line). *p* = 0.0134 based on Poisson regression. Note that the data are plotted with jitter to show individual points, but both copy number and number of infections are integer data. (B) The distribution of *DMBT1* CNV_SRCR9‐11_ copy number in the patients treated with placebo divided between those who had multiple recurrent breakthrough UTIs and those who did not. Multiple (≥2) breakthrough UTIs (black bars) appeared more frequent in patients with *DMBT1* CNV_SRC9‐11_ copy number below the mean (dotted line), while higher *DMBT1* CNV_SRC9‐11_ copy number was associated with a higher prevalence of <2 breakthrough infections. (C) Within the placebo group, patients with “low” (≤5), *DMBT1* CNV_SRC9‐11_ copy number had ∼4‐times the odds of having multiple breakthrough UTIs compared to patient “high” (≥6) *DMBT1* CNV_SRC9‐11_ copy number. No significant increased odds of multiple recurrent UTIs existed between RIVUR patients with “high” and “low” *DMBT1* CNV_SRC9‐11_ copy number in patients treated with antibiotic prophylaxis

Both mice and humans had similar DMBT1/Dmbt1 protein immunolocalization in the urothelium of bladders, kidney, and ureters (Figures 2A and 2B). Wild type (WT) mice had higher bladder but not kidney *Dmbt1* mRNA expression following experimental UTI (Figure 2C). With experimental UTI, bladder counts were 2.4‐fold higher at 6‐h in *Dmbt1^−/‐^* mice compared to WT (*p* = 0.0184) (Figure 2D). No differences were demonstrated in kidney CFU counts at any timepoint or in bladder CFU counts at 24‐h post‐inoculation (Figure 2E). Thus, *Dmbt1* appears to be most important in early time points following bacterial exposure of the lower urinary tract.

**FIGURE 2:**
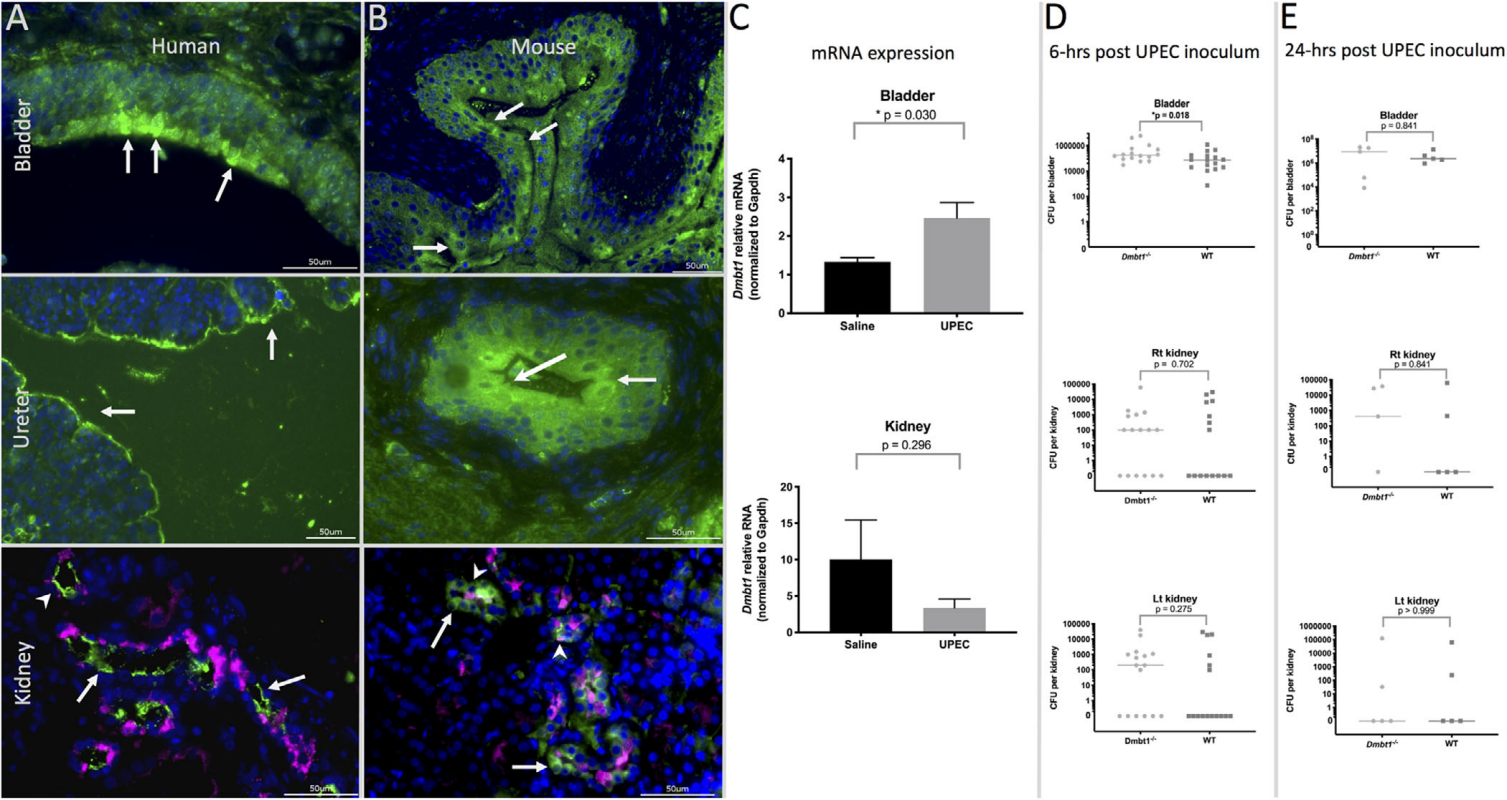
Human (A) and murine urinary (B) tract expression of DMBT1^gp340^ is similar. In human and mouse bladder tissue (A and B, top panel), DMBT1 expression (FITC/green) immunolocalizes to the urothelial cells (white arrows). In the ureter (A and B, middle panel) of humans and mice, DMBT1 (FITC/green) again immunolocalizes to the luminal urothelial cells (white arrows). In the human and mouse kidney (A and B, bottom panel), the collecting duct was localized by V‐ATPase E1 subunit staining (magenta pseudo color of red fluorescence) which labels the collecting duct intercalated cells, while the cells within the collecting duct that do not label with V‐ATPase E1 are presumed collecting duct principal cells. DMBT1^gp340^ expression (FITC/green) occurred in cells of the collecting duct that did not individually label for V‐ATPase E1 (white arrows) consistent with principal cell DMBT1^gp340^ expression. An occasional cell labels for both V‐ATPase E1 and DMBT1^gp340^ (white arrowheads), consistent with some DMBT1^gp340^ expressing intercalated cells. DMBT immunolabeling is apical in distribution in the human ureter (A, middle panel) and kidney principal cells (A, bottom panel, arrows) but is more diffusely cytoplasmic in the mouse ureter (B, middle panel) and kidney principal cells (B, middle panel). (C) *Dmbt1* bladder mRNA expression increases during murine experimental UTI. Bladder *Dmbt1* expression (C, top panel) significantly increased by 1.9‐fold, while kidney *Dmbt1* expression (C, bottom panel) had a nonsignificant trend toward 3.0‐fold decreased *Dmbt1* mRNA expression; *N* = 7 bladders and three kidneys per experimental group (saline vs. UPEC). Data presented as mean ± SEM. Bladder data were analyzed with Welch's *t*‐test (normally distributed but different variances), while the kidney data were analyzed with an unpaired *t*‐test (normally distributed with similar variances). (D) *Dmbt1^−/‐^* mice have increased UTI susceptibility as defined by higher bladder bacterial burdens at 6 h post‐UTI induction. Median bladder CFUs were 2.4‐fold higher (D, top panel), but there were no significant differences in the right (D, middle panel) or left (D, bottom panel) kidney CFUs in *Dmbt1^−/‐^* versus wild‐type mice. At 24‐h (E) there were no differences between bladder (E, top panel) or kidney (E, middle and bottom panels) bacterial CFUs in *Dmbt1^−/‐^* compared to wild‐type mice. Data analyzed with the Mann Whitney test and presented as a scatterplot with median value because the data were not normally distributed. *n* = 16 and five mice per genotype at the 6‐h and 24‐h time points, respectively

To determine if the number of SRCR domains affected UPEC agglutination, we measured the size of bacterial aggregations on plates coated with bovine serum albumin, with both DMBT1 ^gp340^‐short or DMBT1 ^gp340^‐long at different points and concentrations (Figure 3). At the 3‐h time point, significantly larger bacterial agglutinations were noted with DMBT^gp340^‐long compared to DMBT1^gp340^‐short. Both DMBT1^gp340^‐short and DMBT1^gp340^‐long have mild direct antibacterial activity that was similar between the two protein variants (Supplemental material S4).

**FIGURE 3:**
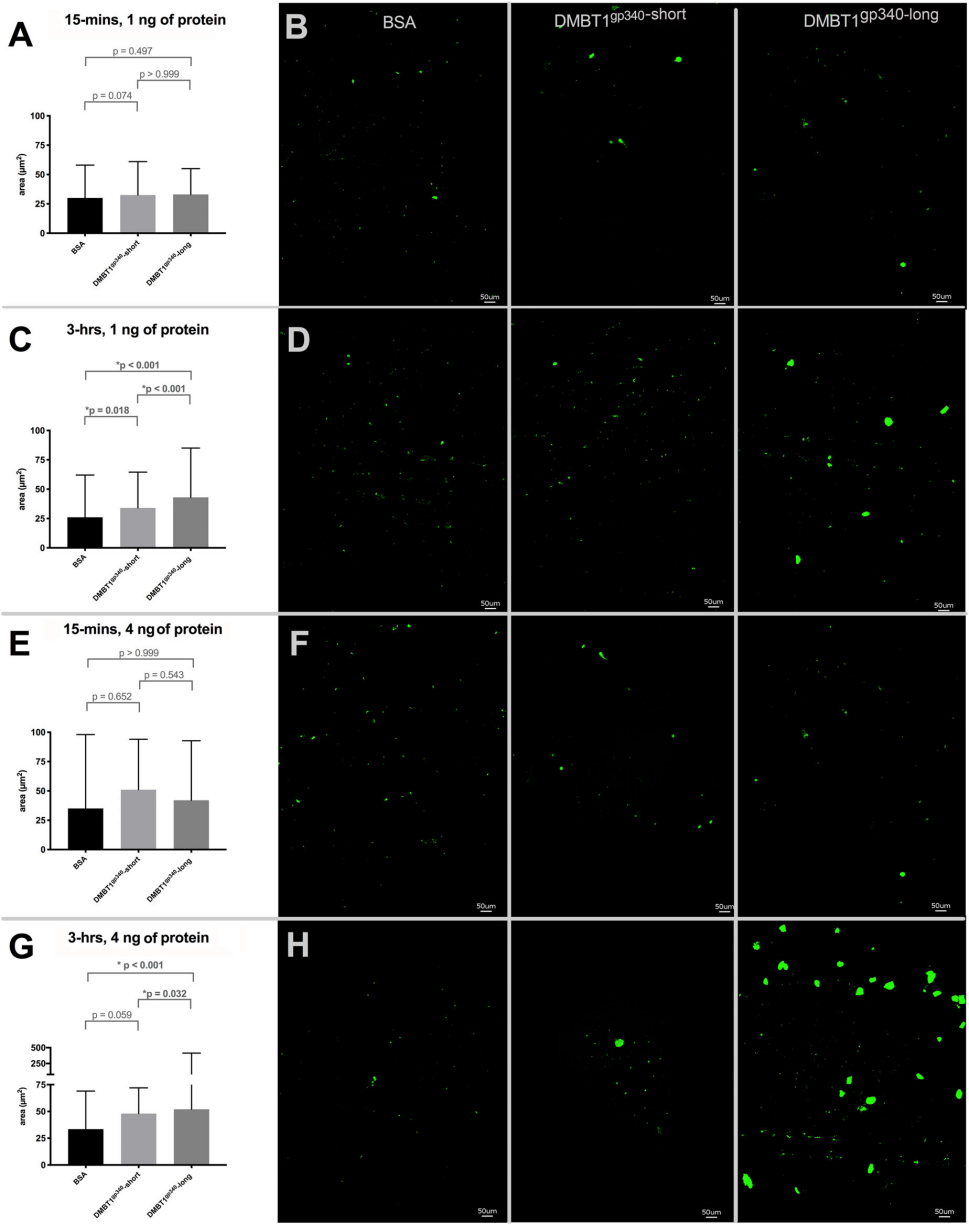
DMBT1^gp340‐long^ protein agglutinates more UPEC than DMBT1^gp340‐short^ protein*. (*A) At 15‐min using 1 ng of protein, there were no differences in the median size of bacterial agglomerations between BSA, DMBT1^gp340‐short^ and DMBT1^gp340‐long^ as visualized in representative images (B). C. At 3‐h using 1 ng of protein, increased mean size of bacterial agglomerations was noted with DMBT1^gp340‐short^ and DMBT1^gp340‐long^ compared to BSA and in DMBT1^gp340‐long^ compared to DMBT1^gp340‐short^ as visualized in representative images (D). (E) At 15‐min using 4 ng of protein, there were no differences in the median size of bacterial agglomerations between BSA, DMBT1^gp340‐short^, and DMBT1^gp340‐long^ as visualized in representative images (F). (G) At 3‐h using 1 ng of protein, increased mean size of bacterial agglomerations was noted with DMBT1^gp340^‐long compared to BSA and in DMBT1^gp340‐long^ compared to DMBT1^gp340^‐^short^ as visualized in representative images (H). The number of bacterial agglomerations (BSA: DMBT1^gp340‐short^: DMBT1^gp340‐long^) measured for analysis were 437:94:87 for 1 ng (15‐min), 391:501:949 for 1 ng (3‐h), 67:83:220 for 4 ng (15‐min) and 152:214:541 for 4 ng (3‐h). The data was non‐parametric, analyzed using the Kruskal‐Wallace test and presented as median ± interquartile range

This study extends our knowledge about DMBT1^gp340^ and *DMBT1* genetic variations in multiple ways (Figure 4). Medication dosing based on an individual's genetic profile is largely limited to drug metabolism genes.^8^ Here, we provide evidence that that genetic variation in innate immune genes may also guide management strategies. Specifically, antibiotic prophylaxis is expected to lower recurrent UTIs in VUR patients with low, but not high *DMBT1* copy number. We also demonstrate increased bladder *Dmbt1* mRNA levels during UTI. We expand on DMBT1's innate immune biological relevance and demonstrate increased bladder UPEC burdens when *Dmbt1* ^−/−^ mice are infected, and increased UPEC agglutination with larger DMBT1 protein size. The *in vitro* agglutination DMBT1 findings are consistent with our *in vivo* murine findings of increased UTI risk at an early 6‐h time‐point and RIVUR patient findings. We hypothesize that the DMBT1 agglutination capability becomes saturated by the later 24‐h time‐point resulting in no increased cystitis.

**FIGURE 4:**
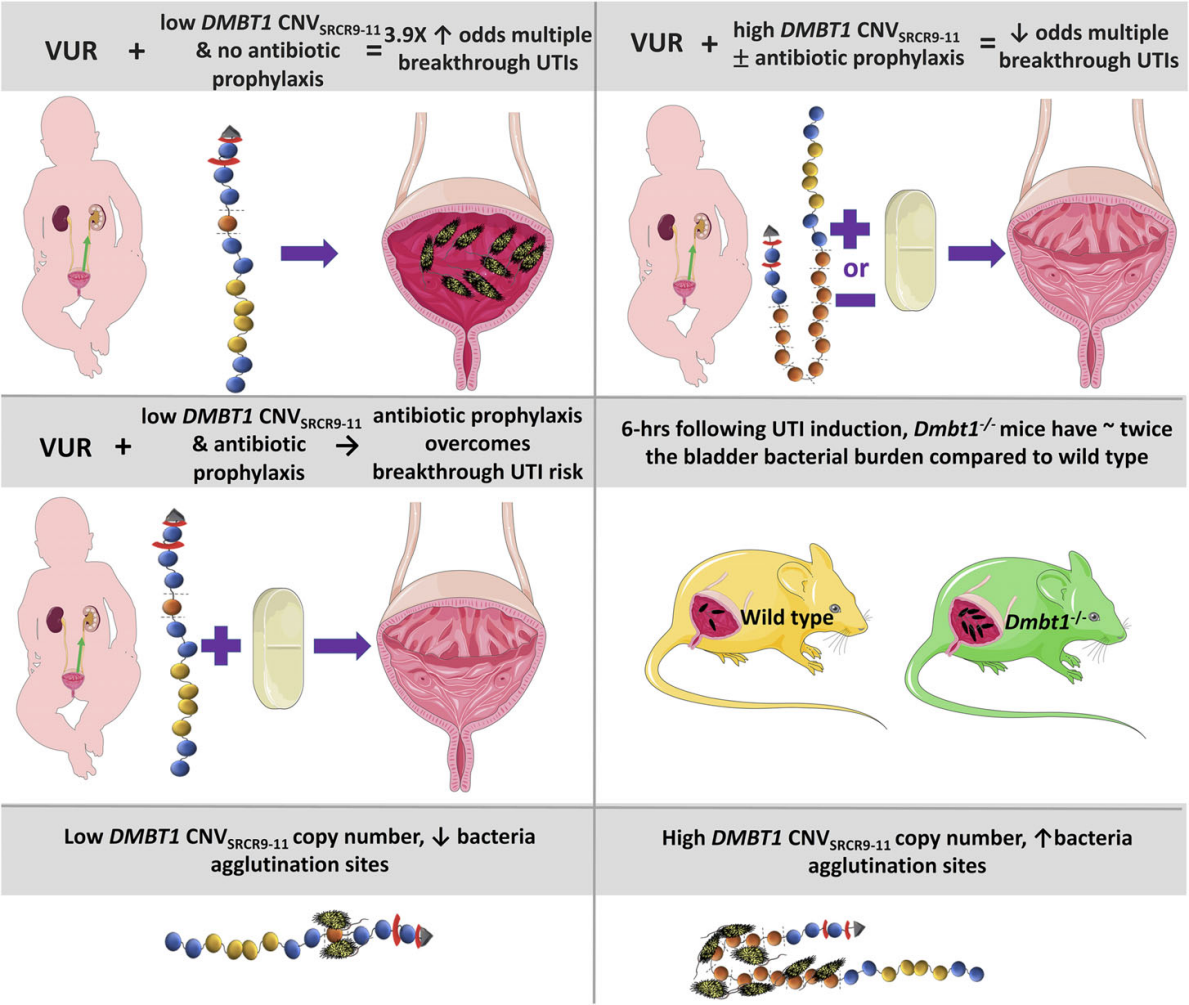
Summary of key findings

This study has a few limitations. DNAs from comparable UTI/VUR treatment cohorts are not currently available for analysis. Secondly, we initially focused on European‐American females because they represent the patients most affected with VUR, for example in the RIVUR cohort, 91.9% of patients were female, and 80.7% were European Americans.^2^ Thus, our results will need to be investigated in males and patients of other races and ethnicities to determine whether they are broadly applicable across patient populations. Our mouse model is a *Dmbt1* knockout, while patients with decreased copy numbers of *DMBT1* CNV_SRCR9‐11_ have a genetic alteration within an otherwise intact *DMBT1* gene.

Our data suggest that genetic variation in innate immune genes such as *DMBT1* copy number warrants investigation as a future tool to guide a clinicians’ decision whether to initiate antibiotic therapy in at‐risk patients.

## CONFLICT OF INTEREST

The authors declare that there is no conflict of interest that could be perceived as prejudicing the impartiality of the research reported.

## DATA AVAILABILITY STATEMENT

The source data are available at the following: https://figshare.com/articles/dataset/DMBT1_experiments_source_data/14208587.

## Supporting information

SUPPORTING INFORMATION
