# Supplementary material for: Deleted in malignant brain tumor 1 genetic variation confers urinary tract infection risk in children and mice
Source: Clin Transl Med. 2021 Jul 4;11(7):e477. doi: 10.1002/ctm2.477 (PMC8255058; doi:10.1002/ctm2.477)
Supplement: Supplementary file 1 — SUPPORTING INFORMATION [file CTM2-11-e477-s001.pdf]

Supplementary materials

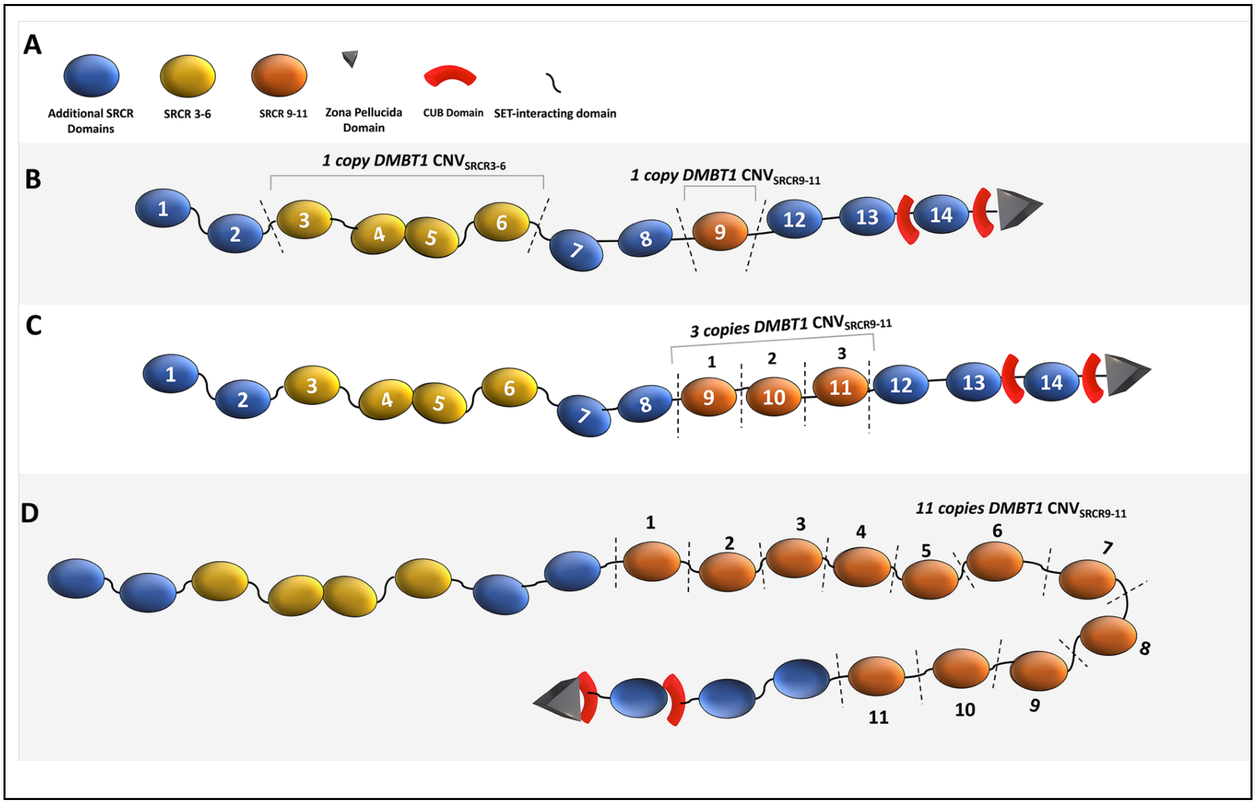

Supplementary material S1. Predicted difference in agglutination potential between copy number range for *DMBT1* $CNV_{SRCR3-6}$ and DMBT1 $CNV_{SRCR9-11}$. (**A**) legend for select DMBT1 domains. (**B**) Representation of 1 copy number each for DMBT1 $CNV_{SRCR3-6 \text{ and}}$ *DMBT1* CNVSRCR9-11. (**C**) Representation for 3 copy number range of DMBT1 $CNV_{SRCR9-11}$. Unlike *DMBT1* CNVSRCR3-6, increased copy number DMBT1 $CNVS_{RCR9-11}$ involves 1 SRCR domain rather than the group of SRCR domains. (**D**). Representation for high range of copy number for *DMBT1* $CNV_{SRCR9-11}$ consistent with more variability in agglutination potential between low and high copy of *DMBT1* $CNV_{SRCR9-11}$. Protein folding is for illustration purposes only and is not meant to represent actual DMBT1 protein folding.

Supplemental material S2: Full methods and acknowledgements

Methods

Study approval

Human subject/sample research was approved by Indiana University Institutional Review Board (IRB) protocol 1709327425 and 1802253259 and Nationwide Children's IRB protocols IRB07-00383 and IRB10-00319. Murine research was approved by Indiana University Institutional Animal Care and Use Committee (IACUC) protocol 11333 and The Research Institute at Nationwide Children's Hospital IACUC protocol AR12-00035. Animal experiments adhered to the "NIH Guide for the Care and Use of Laboratory Animals" and human experiments adhered to the "Declaration of Helsinki."

Patient samples

DNA was obtained from children with UTI and VUR enrolled in the RIVUR Study (ClinicalTrials.gov Identifier NCT00405704). For complete RIVUR study design and outcome data, please refer to previously published manuscripts. The RIVUR study enrolled 607 children aged 1-71 months and documented VUR by voiding cystourethrogram grades I-IV and 1-2 documented UTIs. DNA was generated from lymphoblastic cell lines by the National Institute of Diabetes and Digestive and Kidney Diseases biorepository. Human kidney and ureter tissue was obtained from the Cooperative Human Tissue Network. Human bladder tissue from ureteral reimplantation procedures at Riley Children's Hospital was obtained following written consent. Control DNA was obtained from children at Nationwide Children's Emergency Department who had no history of UTIs, VUR or renal anomalies. Control DNA was collected as saliva samples and stored in Oragene kits (OG-250, DNA Genotek, Ottawa, Canada). Controls were only included if age, race, and sex data were available. Human kidney tissue was from the Cooperative Human Tissue Network (Columbus, OH, www.CHTN.org). Bladder and ureter tissue was obtained from leftover tissue obtained during ureteral reimplantation procedures.

Mice

Wild type C57BL/6 (Stock No. 000664, Jackson Laboratories, Bar Harbor, Maine) and *Dmbt1*$^{-/-}$ mice, previously generated by the Mollenhauer research group, on a C57BL/6 background were used. Female mice between 9-12 weeks of age were used for UTI experiments.

Housing and Husbandry

All mice used for this experiment were bred and kept in a pathogen free housing facility at the Indiana University School of Medicine, Indianapolis, IN. Mice for experiments were all female and kept separated by sex, with no more than female male mice being in a single cage at one time. Bedding material used was wood shavings for the main base with tissue paper also being provided to mice for bedding. *Dmbt1*$^{-/-}$ mice on a C57BL/6 background were bred in a homozygote to homozygote arrangement with 1 male with 2 females under normal, none-altered conditions with water, light, or food.

Animals were weaned from breeding pairs at 28 days and separated in to separate cages depending on date of birth as well as sex. WT C57Bl/6 mice were acclimated to the same vivarium, room, and cage conditions as the *Dmbt1*$^{-/-}$ mice for at least 4 weeks before experimentation.

Bacteria

UPEC strain CFT073 (American Type Culture Collection (ATCC 700928)) was used. GFP expressing UPEC CFT073 was a kind gift from Matthew Mulvey (University of Utah).

Recombinant DMBT1

The ATCC A549 cell line was genetically modified by stable transfection of a plasmid carrying the DMBT1 gene, which stably expressed rDMBT1 6kb (DMBT1^{gp340}-short) or rDMBT1 8kb (DMBT1^{gp340}-long) recombinants under the control of a doxycyclineinducible CMV promoter. DMBT1^{gp340}-short and DMBT1^{gp340}-long were purified by bacterial enrichment followed by fast protein liquid chromatography (FPLC) purification.

DMBT1 copy typing

Copy number at *DMBT1* $CNV_{SRCR3-6}$ or *DMBT1* $CNV_{SRCR9-11}$ was determined using paralog ratio tests (PRT), a form of quantitative PCR. Raw PRT data were clustered into integer copy number estimates and assigned a posterior probability of that integer copy number being correct using a Gaussian mixture model by the CNVtools package in the statistical software R v3.0.

Murine experimental UTI

Female mice were inoculated with UPEC by transurethral injection and, following euthanasia at 6 and 24 hours, the bladder or kidney bacterial burdens were quantified. A UPEC inoculum was 10^8 CFT073 in 50 µl sterile PBS. A second inoculum of the same concentration was performed 3-hours later. Kidney and bladder tissue was also obtained for RT-PCR analysis.

Quantitative real-time PCR

RNA was purified from tissues using RNeasy plus kit (Qiagen, CA), and purity and quantity was measured on a Nanophotometer NP80 (Implen, CA). cDNA was generated. cDNA was amplified using the 7500 Real-Time PCR System (Applied Biosystems, Carlsbad, CA) according to the manufacturer's instructions. Targeted PCR for *Dmbt1* and housekeeping gene *Gapdh* mRNA expression used the primers: *Gapdh*, Forward, 5`-CTGGAGAAACCTGCCAAGTA-3` and Reverse, TGTTGCTGTAGCCGTATTCA and *Dmbt1*, Forward, CATCTGTTCAGCTTCTCAATC and Reverse, TTTGTTCAGTTGGGTAGAAC. Relative *Dmbt1* mRNA expression was quantified using the 2^{-DDCT} methodology.

Immunostaining

Following deparaffinization, rehydration, antigen retrieval, and blocking, slides were incubated with primary antibody overnight at 4°C and incubated for 30 min at room temperature with fluorophore-conjugated secondary antibody for immunofluorescence. Slides were mounted in Vectashield Mounting Media with DAPI (4′, 6-diamidino-2phenylindole) (Vector Laboratories, Burlingame, CA). Imaging was performed with a Keyence BZ-9000 “all in one” microscope and camera (Keyence, Osaka, Japan). Primary antibodies were anti-P84 (Produced in rabbit against $DMBT1^{gp340}$, Mollenhauer lab, Denmark) and anti-ATP6V1E1 (Produced in Chicken, Sigma-Aldrich, St. Louis, MO) for staining of both human and mice. Secondary antibodies consisted of AlexaFlour 488 conjugated anti-Rabbit IgG and Cy3 conjugated anti-Chicken IgG (Jackson ImmunoResearch, West Grove, PA). Keyence BZ analyzer software was used to process the image (brightness, contrast, and black balance).

Bacterial growth study

The antimicrobial activity of recombinant $DMBT^{gp340}$ was evaluated against UPEC using colony count reduction assays. UPEC (strain UTI89) were incubated with serial dilutions of recombinant DMBT1 for 2-hours at 37°C. The antimicrobial activity of $DMBT1^{gp340}$ was analyzed by plating the incubation mixtures and determining the CFUs the following day. Repeat testing was performed on all bacterial isolates in duplicate (n=4).

Aggregation studies

UPEC at a concentration of ~5,000 CFU were incubated with $DMBT1^{gp340}$-short, $DMBT1^{gp340}$-long or control bovine serum albumin (BSA). Bacteria were stained with SYTO9 and ~10µL of bacteria protein suspension was mounted on a slide and imaged with a Keyence BZ-9000 microscope. Bacterial aggregations were identified, and those areas quantified with the “dynamic cell count” and “cell count” features of BZ-II Analyzer software (Keyence) using data from 3-6 images obtained from 2 experiments.

Statistical approach and data analysis

Poisson regression on UTI occurrence by *DMBT1* copy numbers with an adjustment for antibiotics treatment was performed using the open source statistical software Rv.3.0 (www.r-project.org). Low (£ 5), or high (>5) CNVs of *DMBT1* were assessed for association with multiple (³ 2) recurrent UTIs using Fisher's exact test. The Welch two sample t-test was used to compare RIVUR vs control patient *DMBT1* copy number. Data from RT-PCR, kidney and bladder bacterial burden and bacterial aggregation results were analyzed using Graphpad Prism (GraphPad, San Diego, CA). Data was evaluated for normality using the Shapiro Wilk normality test. Parametric data was compared using the t-test if 2 groups were analyzed, and a 1-way ANOVA was used if >2 groups were compared. If variances were significant using an F-test, Welch’s correction was used with the t-test. Nonparametric data was compared using the MannWhitney test if 2 groups were compared, and Kruskal-Wallis test if >2 groups were compared. Since bacterial burdens are presented on a log10 scale, values of 0 were assigned a value of 0.1 for graph generation, but not statistical calculations. Figures

were generated with Powerpoint (Microsoft, Richmond, Washington) and Servier Medical Art (http://www.**servier**.com).

Acknowledgments: We acknowledge Jan Mollenhauer from the University of Southern Denmark whose past work provided the basis for this paper. Additionally his expertise and development of the knockout mice and many of the reagents listed in this study were critical in the successful execution of these experiments. Moreover, we gratefully acknowledge Prof. Mogens Kilian from the Department of Biomedicine of Aarhus University for providing the S. *mutans* bacteria, and Ms. Vicki Nielsen for technical assistance with FPLC. We acknowledge the RIVUR Trial Investigators and the NIDDK for their support. We acknowledge the Cooperative Human Tissue Network for tissues used in this study, Sherry Wilson, RN, for her work in enrolling patients for collection of leftover bladder tissue, and Jennifer Kline and the Nationwide Children's Hospital Emergency Department for enrolling patients for control DNA. The work was supported in part by grants from the United States National Institute of Diabetes and Digestive and Kidney Diseases (R01DK106286, 1RC4DK090937), Lilly Endowment, Inc. Physician Scientist Initiative, Curie ITN-PathChooser (PITN-GA-2013-608373), the Lundbeckfonden Center of Excellence NanoCAN, and the DAWN-2020 project of the SDU Presidents SDU2020 program, a Medical Research Council New Investigator Grant (GO801123), and a PhD studentship from the Government of India Ministry of Social Justice and Empowerment. The RIVUR study was funded by grants U01 DK074059, U01 DK074053, U01 DK074082, U01 DK074064, U01 DK074062 and U01 DK074063 from the NIDDK, National Institutes of Health, Department of Health and Human Services, as well as University of Pittsburgh Clinical and Translational Science Award (UL1RR024153 and UL1TR000005), and the Children's Hospital of Philadelphia Clinical and Translational Science Award (UL1TR000003), both from the National Center for Research Resources, now at the National Center for Advancing Translational Sciences, National Institutes of Health.

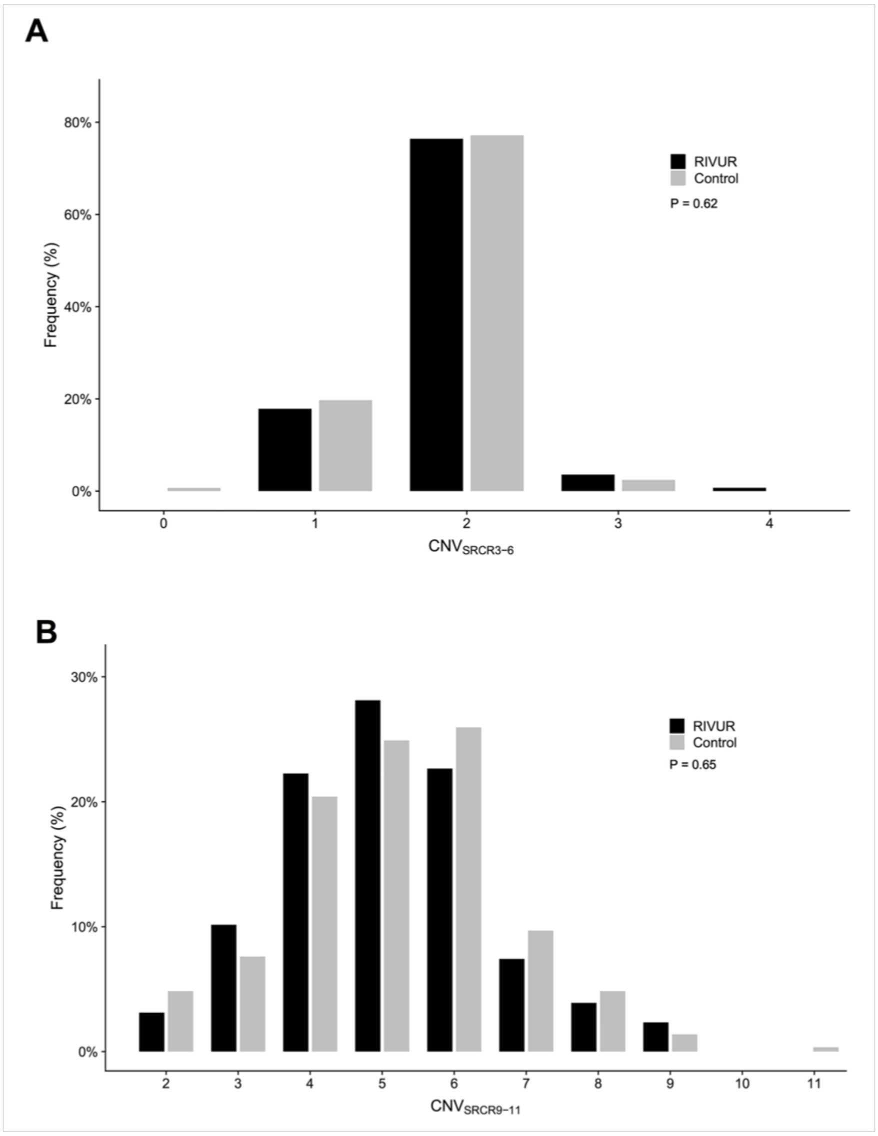

Supplemental material S3: Distribution of *DMBT1* $CNV_{SRC9\text{-}11}$ and $CNV_{SRCR3\text{-}6}$ and in the RIVUR vs control patient distribution of *DMBT1* $CNV_{SRCR3\text{-}6}$ (**A**) and $CNV_{SRC9\text{-}11}$ (**B**) copy numbers in RIVUR patients with and without recurrent UTIs.

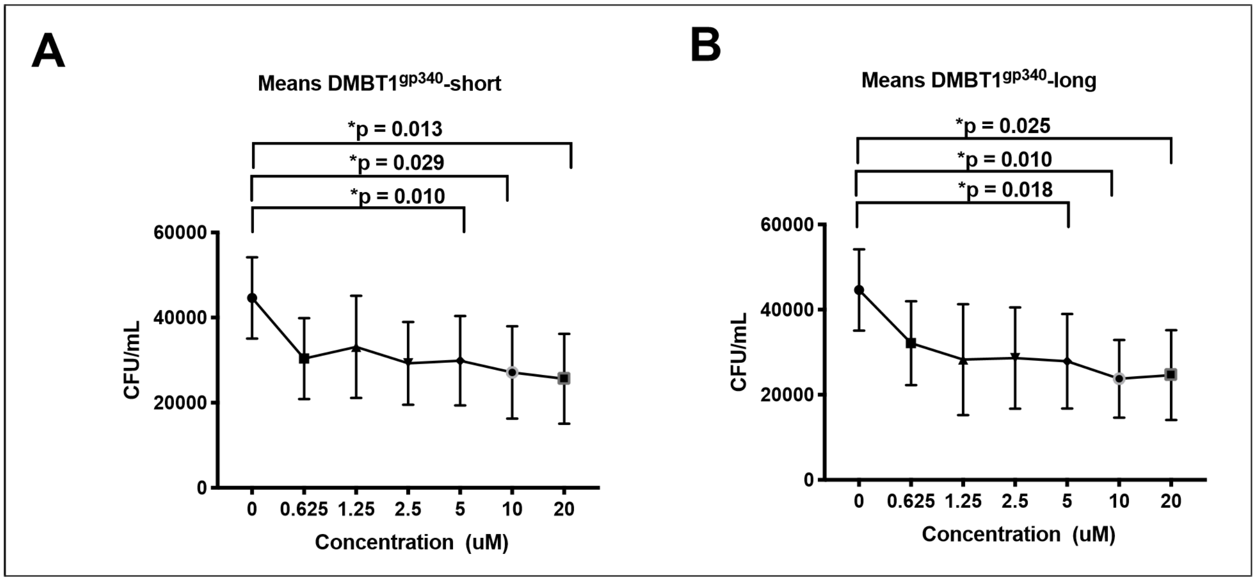

Supplemental material S4: DMBT1^{gp340} protein has antibacterial activity.
(**A**) Compared to baseline (0 mM of DMBT1^{gp340}), DMBT1 ^{gp340}-short does not significantly decrease CFUs/ml of UPEC at 0.625 mM 1.25 mM or 2.5 mM concentrations, but there were significant decreases by a factor of 1.5-fold at 5 mM, 1.6-fold at 10 mM and 1.7-fold at and 20 mM concentrations. (**B**) DMBT1^{gp340}-long also does not significantly decrease CFUs/ml of UPEC at 0.625 mM 1.25 mM or 2.5 mM concentrations, but there were significant decreases by a factor of 1.6-fold at 5 mM, 1.9-fold at 10 mM and 1.8-fold at and 20 mM concentrations. Data analyzed as repeated measures one-way ANOVA and data presented as means ± SEM. Means of 4 independent runs performed in duplicate were evaluated**.**
